# Uremic Pruritus: Prevalence and Impact on Quality of Life and Depressive Symptoms in Hemodialysis Patients

**DOI:** 10.7759/cureus.5178

**Published:** 2019-07-19

**Authors:** Muhammad Zubair Satti, Danish Arshad, Hassan Javed, Ahmad Shahroz, Zeeshan Tahir, Mian Muhammad Hassan Ahmed, Arslan Kareem

**Affiliations:** 1 Medicine, Rawalpindi Medical University and Allied Hospitals, Rawalpindi, PAK; 2 Psychiatry, Rawalpindi Medical University and Allied Hospitals, Rawalpindi, PAK; 3 Internal Medicine, Rawalpindi Medical University and Allied Hospitals, Rawalpindi, PAK

**Keywords:** uremic pruritus, end stage renal disease, depression, quality of life

## Abstract

Background

End-stage renal disease (ESRD) is a major public health problem with many associated symptoms. Uremic pruritus (UP) develops in 40% of patients on hemodialysis and has major effects on the patient’s life. It is also an independent risk factor for increased mortality, and its psychiatric implications remain poorly characterized in our local setup, where it tends to be underdiagnosed and undertreated.

Objectives and rationale

The study aims to report the prevalence of uremic pruritus in our study population and associate it with various patient parameters, which may define a subset of patients at high risk for this pruritus. We also assess the effects of uremic pruritus on the patient’s quality of life (by using the Dermatology Life Quality Index; DLQI) and depressive symptoms (by using the Public Health Questionnaire; PHQ-9).

Materials and methods

It was a descriptive, cross-sectional study conducted in the nephrology unit of the multi-organ failure (MOF) center of the Holy Family Hospital (HFH), Rawalpindi, Pakistan, from February 2019 to June 2019, during which 173 male patients on hemodialysis were selected. Informed consent was taken from patients and other skin-related causes of pruritis were excluded. Uremic pruritus was defined as pruritis lasting for at least three months after the onset of ESRD. The 5-D, PHQ-9, and DLQI questionnaires were used to assess pruritis, depressive symptoms, and quality of life, respectively. Their Cronbach's Alpha values for 73 responses were 0.83, 0.81, and 0.71, respectively. The descriptive analysis was performed using SPSS v23.0 (IBM Corp, Armonk, NY, US). Spearman’s rank-order correlation, independent samples t-test, and one-way analysis of variance (ANOVA) were used to analyze study variables.

Results

The prevalence of uremic pruritus was 49.1%, with many patients having generalized itching. Unemployment and longer disease duration predisposed the patients towards uremic pruritus, as the mean 5-D score in this subset were greater (p<0.05 in the independent samples t-test). The results of one-way ANOVA were significant (p<0.05), indicating higher 5-D scores in worsening categories of depressive symptoms and quality of life. Spearman’s correlation matrix showed that 5-D, PHQ-9, and DLQI scores were strongly correlated with each other.

Conclusions

The prevalence of uremic pruritus among male hemodialysis patients is high, at 49.1%. It significantly contributes to depressive symptoms and a lower quality of life, which are associated with worse prognosis in hemodialysis patients. Thus, a clinician must keep in mind the psychiatric implications of uremic pruritus and treat it effectively to optimize the patient’s medical care.

## Introduction

End-stage renal disease (ESRD), which mandates dialysis, is a burgeoning public health problem with an ever-increasing prevalence throughout the world. An estimated 200 million people are reported to suffer from chronic kidney disease (CKD) and many of these patients eventually progress to ESRD [1-3]. In recent times, many efforts have been made to improve the survival of hemodialysis patients with end-stage renal disease. While hemodialysis has improved the survival rates, concerns regarding a reduction in quality of life (QoL) due to ESRD are worrisome and have been reported by numerous studies [4-5]. Thus, the need for an improvement in the quality of life in chronic hemodialysis patients with end-stage renal disease has been strongly emphasized.

The lower QoL in ESRD is because of many associated life-limiting symptoms such as pruritus. Pruritus associated with ESRD (called uremic pruritus) is an irritating sensation of the skin that causes a desire to scratch. The prevalence of uremic pruritus is reported to vary, affecting around one-third of these patients [6]. Moreover, unlike other symptoms of ESRD, dialysis only has the mild effect of reducing pruritus, and many patients undergoing hemodialysis complain of varying degree of pruritus [7-8]. The extent and severity of uremic pruritus vary between patients. In some patients, it is extensive and persistent while in others, it may be short-lived and localized. Severe uremic pruritus was found to be an independent risk factor of mortality in ESRD patients [9-10].

The exact pathogenesis of uremic pruritus remains unclear, but it is postulated to be multifactorial [11]. Histamine, parathormone, and electrolyte derangements (particularly of calcium and magnesium salts) have been implicated in pruritus and newer hypotheses focus on micro-inflammation and opioid-receptor malfunctions as possible causes of ESRD-associated pruritus [11-12]. There are a variety of therapeutic interventions that are useful in the treatment or reduction of the severity of uremic pruritus, such as correction of calcium and phosphorus levels, phototherapy, and acupuncture, with varying results [13].

Another CKD-associated condition is depressive symptoms with a high reported prevalence in this population [14]. It is associated with poor adherence to dialysis, medications, nutrition, poor quality of life, and higher mortality [14]. We hypothesize that one of many factors responsible for depressive symptoms may be uremic pruritus, as it lowers QoL, makes social interaction difficult, and leads to a continuous nagging sensation of itching along with disturbed sleep. All these possible effects of this pruritis have been linked to depressive symptoms in many studies [15]. However, we found no such study in our local set-up that considers the psychiatric implications of uremic pruritus.

Thus, chronic pruritus represents a serious public health concern that must be adequately addressed by clinicians. Despite the high prevalence and life-changing effects of pruritus in hemodialysis patients, the assessment of its effects on the lives of patients remains poorly characterized, especially in our local settings. Therefore, the goal of this study is to determine the prevalence and impact of uremic pruritus on the patient’s quality of life and to analyze whether it is associated with depressive symptoms in this population. We also assess various patient-related factors for their association with uremic pruritus. The results will help the local physicians to inquire and treat the pruritus in these patients so that their QoL can be improved. Furthermore, the results may also identify a subset of the dialysis population at high risk for uremic pruritus.

## Materials and methods

Study design

It was a descriptive, cross-sectional study conducted in the nephrology unit of the multi-organ failure (MOF) center of Holy Family Hospital (HFH), Rawalpindi, Pakistan, from February 2019 to June 2019. The study population included patients with ESRD on hemodialysis (for at least three months). Randomization was performed by systemic sampling in which patients coming for dialysis were numbered randomly, and each patient with an even number was selected for the study. The mini-mental state examination (MMSE) was used to evaluate the mental state of the patients, and those with a score of less than 19 were excluded from the study, as they may not be able to deliver reliable answers.

It was ensured that the itch reported by the patients was due to uremic pruritis and not because of related skin conditions, such as psoriasis, eczema, or dermatitis, by excluding them one by one. Uremic pruritus was defined as pruritus lasting for at least three months after the onset of ESRD. However, to ensure accurate recall, questions about the intensity and severity of the pruritus were asked, considering only the last two weeks according to the 5-D itch scale. During the study period, 173 male patients were interviewed at the bedside by trained investigators after obtaining informed consent. Only male patients were selected for the study because of their easy availability in the dialysis center of our hospital. Moreover, since males have a greater predisposition towards rapid progression to ESRD and earlier hemodialysis as compared to females, it is imperative to see the factors that affect their QoL [1]. Also, due to cultural norms in Pakistan, women tend to undergo peritoneal dialysis at home, which limits the accessibility to this subset of the population.

Questionnaires used

Multiple questionnaires were employed for the collection of data, including:

*1) 5-D Itch Scale* 

To assess pruritus in the population, we used the 5-D itch scale, which inquires five dimensions related to pruritus: duration, degree, direction, associated disability, and distribution of pruritus. The categories of the 5-D itch scale have been equivalated with the numeric rating scale (NRS) by a study that concludes that for the 5-D scale, scores of less than 9 mean no pruritus, 9-11 indicate mild, 12-17 indicate moderate, and 18-21 indicate severe pruritus [16].

2) Public Health Questionnaire-9

To assess depression, the Urdu version of the Public Health Questionnaire-9 (PHQ-9) was used, which is a nine-item scale with a scoring range of 0-27. Scores from 0-4 correspond to no depression, 5-9 show mild, 10-14 moderate, 15-19 to moderately severe, while scores above 20 are consistent with severe depression [17].

3) Dermatology Life Quality Index (DLQI)

The self-explanatory DLQI questionnaire was used to observe the effects of pruritus on a patient’s quality of life. Its reliability for the purpose is also well-established by many studies [18]. A score of 0-1 means there is no effect of pruritis on the patient’s life, 2-5 indicates small, 6-10 indicates moderate, 11-20 indicates large, and 21-30 means very large and severe limiting effects on the patient’s life due to the associated pruritis.

Cronbach's alpha of the questionnaires

The inter-scale reliability of the questionnaire in our study population was assessed by calculating Cronbach's alpha for the first 73 responses. It was 0.83 for the 5-D itch scale, 0.81 for PHQ-9, and 0.71 for DLQI, indicating the high reliability of these questionnaires in our study population.

Statistical analysis

A total of 173 male patients constituted our final sample for an alpha value of 0.05 and power of study at 80%. A descriptive analysis of the study variables was performed using SPSS v23.0 (IBM Corp, Armonk, NY, US). The normality of the data was checked by the Shapiro Wilk test. We assessed the population parameters that were significantly associated with the 5-D itch scale by the independent samples t-test (where means were compared) and Spearman’s rank-order correlation (where continuous variables were available). Then, we analyzed the difference in mean 5-D itch scores regarding different categories of PHQ-9 and DLQI questionnaires by one-way analysis of variance (ANOVA). Further, since the scores were non-normally distributed, continuous variables, a correlation matrix was drawn using Spearman’s rank-order correlation to observe the correlation between the 5-D, PHQ-9, and DLQI scores. A p-value of less than 0.05 was considered statistically significant.

## Results

Population demographics and parameters are provided in Table 1. The table also shows the parameters that are significantly associated with the 5-D itch scale by appropriate tests of significance.

**Table 1:** Demographic details and their association with the 5-D score

Parameters		Test of significance with 5-D scores	p-values
Employment (n=173)	Mean 5-D scores		
Employed=116 (67.1%)	9.2±1.7	Independent samples t-test	p=0.01
Unemployed=57 (32.9%)	11.8±3,2		
Session timing (n=173)			
Morning session=68 (39.3%)	10.7±2.5	Independent samples t-test	p=0.53
Evening session=105 (60.7%)	11.1±2.9		
Age=39.4±4.3		Spearman’s correlation r=0.14	p=0.45
Disease years=5.9±2.8		Spearman’s correlation r=0.64	p=0.002
Hemodialysis years=2.1±1.2		Spearman’s correlation r=0.12	p=0.63

Table 2 shows the details of pruritus in the population. The distribution of pruritus among patients was significant for generalized pruritis.

**Table 2:** Details of pruritus in the population DLQI: Dermatology Life Quality Index; PHQ-9: Public Health Questionnaire

Parameter	Number of patients
Mean 5-D score of the population (n=173)	10.9±2.3
Pruritus intensity (n=173)	
No pruritus	88 (50.9%)
Mild	47 (27.2%)
Moderate	29 (16.8%)
Severe	9 (5.2%)
Distribution of pruritus among 85 pruritic patients	
Arms	13 (15.3%)
Legs	12 (14.1%)
Face	11 (12.9%)
Trunk and back	15 (17.6%)
Generalized (2 or more areas)	34 (40.0%)
Mean DLQI score (n=85)	9.8±1.7
Mean PHQ-9 score (n=173)	12.2±2.9
Chi-square test for the distribution of pruritis was significant at p=0.02	

Table 3 shows the distribution of 5-D scores in different categories of PHQ-9 and DLQI scores. The scores increase in higher degrees of depressive symptoms or lifestyle limitation, which was statistically significant, as means scores had p<0.05 for both indices when calculated by one-way analysis of variance (one-way ANOVA).

**Table 3:** 5-D scores in different categories of PHQ-9 and DLQI scores DLQI: Dermatology Life Quality Index; PHQ-9: Public Health Questionnaire; ANOVA: Analysis of Variance

Parameter	PHQ-9 categories (n=173)					One-way ANOVA (p-value)
	No depression	Mild	Moderate	Moderately severe	Severe	
Number of patients	106 (61.3%)	31 (17.9%)	21 (12.1%)	12 (6.9%)	3 (1.7%)	
5-D itch score	8.1±1.8	10.7±2.5	13.1±3.7	17.6±4.8	19.5±5.8	0.001
DLQI categories (n=85)						
Parameter	No effect	Small	Moderate	Large	Extremely large	One-way ANOVA (p-value)
Number of patients	16 (18.8%)	20 (23.5%)	29 (34.1%)	14 (16.5%)	6 (7.1%)	
5-D itch score	10.2±2.7	12.7±3.2	15.1±4.7	18.3±4.9	20.2±5.6	0.005

Table 4 shows the correlation matrix between the 5-D, PHQ-9, and DLQI scores drawn by using Spearman’s rank-order correlation. 

**Table 4:** Correlation matrix by Spearman's correlation DLQI: Dermatology Life Quality Index; PHQ-9: Public Health Questionnaire

Parameter	Spearman’s coefficient (r) and p-values		
	5-D itch score	DLQI scores	PHQ-9 scores
5-D itch score	1	r=0.78, p <0.000	r=0.66, p=0.01
DLQI scores	r=0.78, p <0.000	1	r=0.69, p=0.01
PHQ-9 scores	r=0.66, p=0.01	r=0.69, p=0.01	1

## Discussion

The results of our study provide important insights into the problem of uremic pruritus among male hemodialysis patients. We found a prevalence of 49.1% (85 out of 173) regarding uremic pruritus, where the majority of the patients had mild to moderate degrees of pruritus as classified by the 5-D itch scale. The prevalence is similar to what other studies reported and indicates the widespread occurrence of uremic pruritus in dialysis patients [19-21]. Many patients (40%) reported generalized pruritic itching involving their entire bodies, which were characteristically more severe on the face and back. Other patients (60%) said that itching was localized and the most common location was the trunk and back for localized itching sensation. Similar results were reported by another study, which also found that in many patients, uremic pruritus presented as generalized itching and discomfort among patients [7].

When we analyzed the association between uremic pruritus and patient parameters, we found some important associations with uremic pruritus. In our study, those males who were employed had significantly lower 5-D scores than unemployed males (p-value of t-test was 0.01). As employment keeps a person busy, the lower 5-D scores might relate to the psychological masking effect of a regular job, which may itself suppress the desire to itch. We also found that disease years had a moderate correlation with 5-D scores, at significant p-values. Since it is the decline in kidney function that contributes to uremic pruritus, more disease years will automatically translate to worsening kidney function and, hence, higher 5-D scores. The results are important and agree with another study conducted in Pakistan [22]. Our study indicates that age, hemodialysis years, and timing of dialysis session had no significant association with 5-D scores, which is in agreement with previous studies [20,23].

Next, we assess the distribution of 5-D scores in different categories of depression and lifestyle limitation. To have a good outlook of the effects of uremic pruritus, only the DLQI questionnaires of patients who had at least nine points in the 5-D scale were included in the analysis. This allowed us to measure QoL, particularly in association with uremic pruritus. No such modification was made for depressive symptoms because they have multifactorial causation. Our results indicate that depressive symptoms and lifestyle limitations are strongly correlated with 5-D scores and a significant ANOVA test concludes that higher degrees of PHQ-9 and DLQI have higher mean 5-D scores. A patient with higher 5-D scores, indicating worsening pruritus also suffers from a greater magnitude of depressive symptoms and lower QoL proportionately. This has an important clinical manifestation because a clinician must tend to treat pruritus in these patients by medical or non-medical measures instead of just waiting to see if it resolves with hemodialysis. Lower QoL and depressive symptoms have been linked to a lack of motivation, poor compliance with medical therapy, and higher mortality rates among hemodialysis patients [24]. Since both are significantly correlated with 5-D scores, a physician must always keep in mind the appearance of pruritus and its depressive effects in all patients of ESRD. These findings agree with many previous studies and underscore the grave consequences of untreated pruritis in hemodialysis patients [23,25-26].

Uremic pruritus generally remains undertreated in many dialysis patients and only routine antihistamines are prescribed, especially in developing countries [24,27]. By highlighting the importance of uremic pruritus in association with depressive symptoms and lower quality of life, our study calls for immediate and effective treatment of pruritis in hemodialysis patients so that their medical care is optimized and quality of life is improved.

## Conclusions

The prevalence of uremic pruritus among male patients on hemodialysis was 49.1%. The pruritus was generalized in many patients, and unemployed males with longer disease years were more prone to suffer from this pruritus. The 5-D score was strongly correlated with depressive symptoms and lower QoL among subjects, indicating that uremic pruritus contributes to depression and lifestyle limitations. These findings mandate the need for the immediate and effective treatment of uremic pruritus among dialysis patients so that their mortality is reduced and quality of life is enhanced.
